# Fast and robust group-wise eQTL mapping using sparse graphical models

**DOI:** 10.1186/s12859-014-0421-z

**Published:** 2015-01-16

**Authors:** Wei Cheng, Yu Shi, Xiang Zhang, Wei Wang

**Affiliations:** 1 Department of Computer Science, UNC at Chapel Hill, 201 S Columbia St., Chapel Hill, 27599 NC USA; 2 Computer Science at the University of Illinois at Urbana-Champaign, 201 North Goodwin Avenue, Urbana, 61801 IL USA; 3 Department of Elect. Eng. and Computer Science, Case Western Reserve University, 10900 Euclid Avenue, Cleveland, 44106 OH USA; 4 Department of Computer Science, University of California, Los Angeles, 3531-G Boelter Hall, Los Angeles, 90095 CA USA

**Keywords:** eQTL mapping, Group-wise association, Sparse graphical model

## Abstract

**Background:**

Genome-wide expression quantitative trait loci (eQTL) studies have emerged as a powerful tool to understand the genetic basis of gene expression and complex traits. The traditional eQTL methods focus on testing the associations between individual single-nucleotide polymorphisms (SNPs) and gene expression traits. A major drawback of this approach is that it cannot model the joint effect of a set of SNPs on a set of genes, which may correspond to hidden biological pathways.

**Results:**

We introduce a new approach to identify novel *group-wise* associations between sets of SNPs and sets of genes. Such associations are captured by hidden variables connecting SNPs and genes. Our model is a linear-Gaussian model and uses two types of hidden variables. One captures the set associations between SNPs and genes, and the other captures confounders. We develop an efficient optimization procedure which makes this approach suitable for large scale studies. Extensive experimental evaluations on both simulated and real datasets demonstrate that the proposed methods can effectively capture both individual and group-wise signals that cannot be identified by the state-of-the-art eQTL mapping methods.

**Conclusions:**

Considering group-wise associations significantly improves the accuracy of eQTL mapping, and the successful multi-layer regression model opens a new approach to understand how multiple SNPs interact with each other to jointly affect the expression level of a group of genes.

**Electronic supplementary material:**

The online version of this article (doi:10.1186/s12859-014-0421-z) contains supplementary material, which is available to authorized users.

## Background

Expression quantitative trait loci (eQTL) mapping is the process of identifying single nucleotide polymorphisms (SNPs) that play important roles in the expression of genes. It has been widely used to dissect genetic basis of complex traits [1,2]. Traditionally, associations between individual expression traits and SNPs are assessed separately [3,4].

Since genes in the same biological pathway are often co-regulated and may share a common genetic basis [5,6], it is crucial to understand how multiple modestly-associated SNPs interact to influence the phenotypes [7]. To address this issue, several approaches have been proposed to study the joint effect of multiple SNPs by testing the association between a set of SNPs and a gene expression trait. A straightforward approach is to follow the gene set enrichment analysis (GESA) [8]. In [9], the authors propose variance component models for SNP set testing. Aggregation-based approaches such as collapsing SNPs are investigated in [10]. In [11], the authors take confounding factors into consideration.

Despite their success, these methods have two common limitations. First, they only study the association between a set of SNPs and a single expression trait, thus overlook the joint effect of a set of SNPs on the activities of a set of genes, which may act and interact with each other to achieve certain biological function. Second, the SNP sets used in these methods are usually taken from known pathways. However, the existing knowledge on biological pathways is far from being complete. These methods cannot identify unknown associations between SNP sets or gene sets.

To address these limitations, in [12], a method is developed to identify cliques in a bipartite graph derived from the eQTL data. Cliques are used to model the hidden correlations between SNP sets and gene sets. However, this method needs the progeny strain information, which is used as a bridge for modeling the eQTL association graphs. In [13], the authors proposed a method to infer associations between sets of SNPs and sets of genes. However, this method does not consider the associations between individual SNPs and genes. A two-graph-guided multi-task Lasso approach was developed in [14]. This method needs to calculate gene co-expression network and SNP correlation network first. Errors and noises in these two networks may introduce bias in the final results. A graph regularized dual lasso approach considering the factor of group-wise association was developed in [15]. This method, however, needs extra SNP-SNP interaction network and PPI network data to penalize the regression model and it’s not able to infer novel group-wise associations. Note that all these methods do not consider confounding factors.

To better elucidate the genetic basis of gene expression and understand the underlying biology pathways, it is highly desirable to develop methods that can automatically infer associations between a group of SNPs and a group of genes. We refer to the process of identifying such associations as *group-wise* eQTL mapping. In contrast, we refer to the process of identifying associations between individual SNPs and genes as *individual* eQTL mapping. In this paper, we introduce a fast and robust approach to identify novel associations between sets of SNPs and sets of genes. Our model is a multi-layer linear-Gaussian model and uses two different types of hidden variables: one capturing group-wise associations and the other capturing confounding factors [11,16-20]. We apply an *ℓ*_1_-norm on the parameters [3,21], which yields a sparse network with a large number of association weights being zero [22]. We develop an efficient optimization procedure that makes this approach suitable for large-scale studies^a^. Extensive experimental evaluations using both simulated and real datasets demonstrate that the proposed methods can effectively capture both group-wise and individual associations and significantly outperforms the state-of-the-art eQTL mapping methods.

## Methods

### Preliminaries

Throughout the paper, we assume that, for each sample, the SNPs and genes are represented by column vectors. Let **x** = [ *x*_1_,*x*_2_,…,*x*_*K*_]^T^ represent the *K* SNPs in the study, where *x*_*i*_∈{0,1,2} is a random variable corresponding to the *i*-th SNP^b^. Let **z**= [ *z*_1_,*z*_2_,…,*z*_*N*_]^T^ represent the *N* genes in the study, where *z*_*j*_ is a continuous random variable corresponding to the *j*-th gene. Table 1 summarizes the main symbols used in this paper.

**Table 1:** **Summary of notations**

**Symbols**	**Description**
*K*	Number of SNPs
*N*	Number of genes
*D*	Number of samples
**x**	The random variables of *K* SNPs
**z**	The random variables of *N* genes
**s**	The latent variables to model confounding
	factors
**y**	The latent variables to model group-wise
	associaiton
$\mathbf {X}\in {\mathbb {R}}^{K \times D}$	The SNP matrix data
*M*	Number of latent variables **y**
*H*	Number of latent variables **s**
$\mathbf {Z} \in {\mathbb {R}}^{N \times D}$	The gene expression matrix data
$\textbf {A} \in \mathbb {R}^{M\times K}$	The coefficient matrix between **x** and **y**
$\textbf {B} \in \mathbb {R}^{N\times M}$	The coefficient matrix between **y** and **z**
$\textbf {C} \in \mathbb {R}^{N\times K}$	The coefficient matrix between **x** and **y**
$\textbf {W} \in \mathbb {R}^{N\times H}$	The coefficient matrix of confounding factors
$\mathbf {\boldsymbol {\mu }_{\textbf {A}}}\in \mathbb {R}^{M\times 1}$, $\boldsymbol {\mu }_{\textbf {B}}\in \mathbb {R}^{N\times 1}$	The translation factor vectors

The traditional linear regression model for association mapping between **x** and **z** is

(1) $$ \mathbf{z}=\boldsymbol{\beta}\mathbf{x}+\boldsymbol{\mu}+\boldsymbol{\epsilon},  $$

where **z** is a linear function of **x** with coefficient matrix ***β***. ***μ*** is an *N*×1 translation factor vector. ***ε*** is the additive noise of Gaussian distribution with zero-mean and variance *ψ* **I**, where *ψ* is a scalar. That is, ***ε***∼*N*(**0**,*ψ* **I**).

The question now is how to define an appropriate objective function to decompose ***β*** which (1) can effectively detect both individual and group-wise eQTL associations, and (2) is efficient to compute so that it is suitable for large-scale studies. In the next, we will propose a group-wise eQTL detection method first, then improve it to capture both individual and group-wise associations. Then we will discuss how to boost the computational efficiency.

### Graphical model for group-wise eQTL mapping

**Figure 1:**
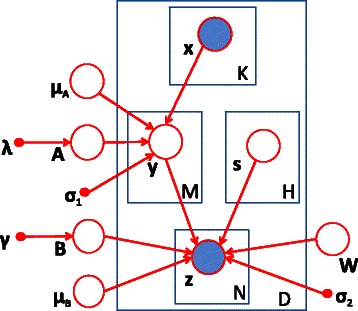
**Graphical model with two types of hidden variables, shaded nodes denote observed random variables, and unshaded nodes denote latent variables.**

To infer associations between SNP sets and gene sets while taking into consideration confounding factors, we propose a graphical model as shown in Figure 1. This model is a two-layer linear Gaussian model. There are two different types of hidden variables in the middle layer. One is used to capture the group-wise association between SNP sets and gene sets. These latent variables are presented as **y**= [ *y*_1_,*y*_2_,…,*y*_*M*_]^T^, where *M* is the total number of latent variables bridging SNP sets and gene sets. Each hidden variable may represent a latent factor regulating a set of genes, and its associated genes may correspond to a set of genes in the same pathway or participating in certain biological function. Another type of hidden variable, **s**= [ *s*_1_,*s*_2_,…,*s*_*H*_]^T^, is used to model confounding factors. Note that this model allows a SNP or gene to participate in multiple (SNP set, gene set) pairs. This is reasonable because SNPs and genes may play different roles in multiple biology pathways.

### Incorporating individual effect

**Figure 2:**
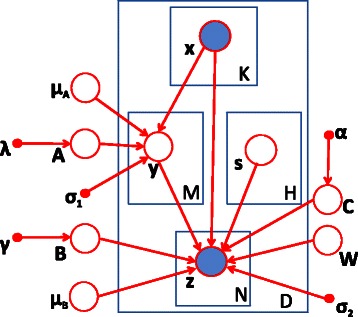
**Refined graphical model to capture both individual and group-wise associations, shaded nodes denote observed random variables, and unshaded nodes denote latent variables.**

In the graphical model shown in Figure 1, we use a hidden variable *y* as a bridge between a SNP set and a gene set to capture the group-wise effect. In addition, individual effects may exist as well [11]. To incorporate both individual and group-wise effects, we extend the model in Figure 1 and add one edge between **x** and **z** to capture individual associations as shown in Figure 2. We will show that this refinement will significantly improve the accuracy of model and enhance its computational efficiency.

### Objective function

Next, we give the derivation of the objective function for the model in Figure 2. We assume that the two conditional probabilities follow normal distributions:

(2) $$ \mathbf{y}|\mathbf{x} \sim N\left(\mathbf{y}|\textbf{A}x+\boldsymbol{\mu}_{\textbf{A}},{\sigma_{1}^{2}}\mathbf{I}_{\mathbf{M}}\right),  $$

and

(3) $$ \mathbf{z}|\mathbf{y,x}\sim N\left(\mathbf{z}|\mathbf{By} + \mathbf{Cx} + \mathbf{Ws} + \boldsymbol{\mu}_{\mathbf{B}},{\sigma_{2}^{2}}\mathbf{I}_{\mathbf{N}}\right),  $$

where $\textbf {A} \in \mathbb {R}^{M\times K}$ is the coefficient matrix between **x** and **y**, $\textbf {B} \in \mathbb {R}^{N\times M}$ is the coefficient matrix between **y** and **z**, $\textbf {C} \in \mathbb {R}^{N\times K}$ is the coefficient matrix between **x** and **z** to capture the individual associations, $\textbf {W} \in \mathbb {R}^{N\times H}$ is the coefficient matrix of confounding factors. $\boldsymbol {\mu }_{\textbf {A}}\in \mathbb {R}^{M\times 1}$ and $\boldsymbol {\mu }_{\textbf {B}}\in \mathbb {R}^{N\times 1}$ are the translation factor vectors, ${\sigma _{1}^{2}}\mathbf {I}_{M}$ and ${\sigma _{2}^{2}}\mathbf {I}_{N}$ are the variances of the two conditional probabilities respectively (*σ*_1_ and *σ*_2_ are constant scalars and **I**_*M*_ and **I**_*N*_ are identity matrices).

Since the expression level of a gene is usually affected by a small fraction of SNPs, we impose sparsity on **A**, **B** and **C**. We assume that the entries of these matrices follow Laplace distributions:

**A**_*i*,*j*_∼**Laplace**(0,1/*λ*),

**B**_*i*,*j*_∼**Laplace**(0,1/*γ*), and

**C**_*i*,*j*_∼**Laplace**(0,1/*α*).

*λ*, *γ* and *α* will be used as parameters in the objective function. The probability density function of **Laplace**(*μ*,*b*) distribution is $f(x|\mu,b)=\frac {1}{2b}\exp \left (-\frac {|x-\mu |}{b}\right)$.

Thus, we have

(4) $$ \mathbf{y} = \mathbf{Ax} + \boldsymbol{\mu}_{\mathbf{A}} + \boldsymbol{\epsilon}_{1},  $$

(5) $$ \mathbf{z} = \mathbf{By} + \mathbf{Cx} + \mathbf{Ws} + \boldsymbol{\mu}_{\mathbf{B}} + \boldsymbol{\epsilon}_{2},  $$

where $\boldsymbol {\epsilon }_{1} \sim N\left (\textbf {0}, {\sigma _{1}^{2}} \mathbf {I}_{M}\right), \boldsymbol {\epsilon }_{2} \sim N\left (\textbf {0}, {\sigma _{2}^{2}} \mathbf {I}_{N}\right)$. From Eq. (2) we have

(6) $$ \mathbf{By}|\mathbf{x} \sim N\left(\mathbf{BAx} + \mathbf{B}\boldsymbol{\mu}_{\mathbf{A}}, {\sigma_{1}^{2}} \mathbf{BB}^{\mathrm{T}}\right),  $$

Assuming that the confounding factors follow normal distribution [11], **s**∼*N*(**0**,**I**_*H*_), then we have

(7) $$ \mathbf{W}\mathbf{s} \sim N(\mathbf{0}, \mathbf{WW}^{\mathrm{T}}).  $$

We substitute Eq. (6), (7) into Eq. (5), and get

$$ {}\mathbf{z}|\mathbf{x}\!\sim\! N\!\left(\mathbf{BAx}\,+\,\mathbf{B}\boldsymbol{\mu}_{\mathbf{A}} \,+\, \mathbf{Cx} \,+\, \boldsymbol{\mu}_{\mathbf{B}}, {\sigma_{1}^{2}} \mathbf{BB}^{\mathrm{T}} + \mathbf{WW}^{\mathrm{T}} \,+\, {\sigma_{2}^{2}} \mathbf{I}_{N}\right). $$

From the formula above, we observe that the summand **B** ***μ***_**A**_ can also be integrated in ***μ***_**B**_. Thus to simplify the model, we set ***μ***_**A**_=**0** and obtain

$$ \mathbf{z}|\mathbf{x} \sim N\left(\mathbf{BAx} + \mathbf{Cx} + \boldsymbol{\mu}_{\mathbf{B}}, {\sigma_{1}^{2}} \mathbf{BB}^{\mathrm{T}} + \mathbf{WW}^{\mathrm{T}} + {\sigma_{2}^{2}} \mathbf{I}_{N}\right). $$

To learn the parameters, we can use MLE (Maximize Likelihood Estimation) or MAP (Maximum a posteriori). Then, we get the likelihood function as $p(\mathbf {z}|\mathbf {x})=\prod _{d=1}^{D} p(\mathbf {z}_{d}|\mathbf {x}_{d})$. Maximizing the likelihood function is identical to minimizing the negative log-likelihood. Here, the negative log-likelihood (loss function) is

(8) $$ \begin{aligned} \mathcal{J}&=\sum_{d=1}^{D}\mathcal{J}_{d}\\ &=-1\cdot\log \prod_{d=1}^{D} p(\mathbf{z}_{d}|\mathbf{x}_{d})\\ &=\sum_{d=1}^{D}(-1)\cdot\log p(\mathbf{z}_{d}|\mathbf{x}_{d})\\ &= \frac{D \cdot N}{2} \log(2 \pi) + \frac{D}{2} \log|\boldsymbol{\Sigma}| \\ &\quad+ \frac{1}{2} \sum_{d=1}^{D} \left[\left(\mathbf{z}_{d} - \boldsymbol{\mu}_{d}\right)^{\mathrm{T}} \boldsymbol{\Sigma}^{-1} \left(\mathbf{z}_{d} - \boldsymbol{\mu}_{d}\right)\right], \end{aligned}  $$

where

$$ \boldsymbol{\mu}_{d} = \mathbf{B A x}_{d} + \mathbf{C} \mathbf{x}_{d} + \boldsymbol{\mu}_{\mathbf{B}}, $$

$$ \boldsymbol{\Sigma} = {\sigma_{1}^{2}} \mathbf{B B}^{\mathrm{T}} + \mathbf{WW}^{\mathrm{T}} + {\sigma_{2}^{2}} \mathbf{I}_{N}. $$

Moreover, taking into account the prior distributions of **A**, **B** and **C**, we have

(9) $$ \begin{aligned} &p(\mathbf{z}_{d},\textbf{A},\textbf{B},\textbf{C}|\mathbf{x}_{d},\textbf{W}, \sigma_{1}, \sigma_{2})\\&\quad= \exp(-\mathcal{J}_{d})\cdot\frac{\lambda}{2}\prod_{i,j}\exp(-\lambda|\textbf{A}_{i,j}|)\\ &\qquad\cdot\frac{\gamma}{2}\prod_{i,j}\exp(-\gamma|\textbf{B}_{i,j}|)\cdot\frac{\alpha}{2}\prod_{i,j}\exp(-\alpha|\textbf{C}_{i,j}|). \end{aligned}  $$

Thus, we have the *ℓ*_1_-regularized objective function

$$\begin{array}{@{}rcl@{}} \max_{\textbf{A},\textbf{B},\textbf{C},\textbf{W},\sigma_{1},\sigma_{2}}\log\prod_{d=1}^{D} p(\mathbf{z}_{d},\textbf{A},\textbf{B},\textbf{C}|\mathbf{x}_{d},\textbf{W}, \sigma_{1}, \sigma_{2}), \end{array} $$

which is identical to

(10) $${} \min_{\textbf{A},\textbf{B},\textbf{C},\textbf{W},\sigma_{1},\sigma_{2}} [\!\mathcal{J}+D\cdot(\lambda||\textbf{A}||_{1}+\gamma||\textbf{B}||_{1}+\alpha||\textbf{C}||_{1})],  $$

where ||·||_1_ is the *ℓ*_1_-norm. *λ*, *γ* and *α* are the *precision* of the prior Laplace distributions of **A**, **B** and **C** respectively. They serve as the regularization parameters and can be determined by cross or holdout validation.

The explicit expression of ***μ***_**B**_ can be derived as follows. When **A**, **B** and **C** are fixed, we have $ \mathcal {J} = \frac {D \cdot N}{2} \log (2 \pi) + \frac {D}{2} \log |\boldsymbol {\Sigma }| + \frac {1}{2} \sum _{d=1}^{D} \vphantom {\boldsymbol {\Sigma }^{-1}}[\!(\mathbf {z}_{d} - \mathbf {B A x}_{d} - \mathbf {C x}_{d}- \boldsymbol {\mu }_{\mathbf {B}})^{\mathrm {T}} \boldsymbol {\Sigma }^{-1} (\mathbf {z}_{d} - \mathbf {B A x}_{d} - \mathbf {C x}_{d} - \boldsymbol {\mu }_{\mathbf {B}})] $. When *D*=1, this is a classic maximum likelihood estimatation problem, and we have ***μ***_**B**_=**z**_*d*_−**B** **A** **x**_*d*_−**C** **x**_*d*_. When *D*>1, leveraging the fact that ***Σ*** ^−1^ is symmetric, we convert the problem into a least-square problem, which leads to

$$\boldsymbol{\mu}_{\mathbf{B}} = \frac{1}{D} \sum\limits_{d=1}^{D} (\mathbf{z}_{d} - \mathbf{B A x}_{d} - \mathbf{C x}_{d}). $$

Substituting it into Eq. (8), we have

(11) $$  \begin{aligned} \mathcal{J} =&\, \frac{D \cdot N}{2} \log(2 \pi) + \frac{D}{2} \log|\boldsymbol{\Sigma}| + \frac{1}{2} {\sum\nolimits}_{d=1}^{D} \left\{\left[\left(\mathbf{z}_{d} - \bar{\mathbf{z}}\right) \right.\right.\\&\left.- \left(\mathbf{B A} + \mathbf{C}\right) \left(\mathbf{x}_{d} - \bar{\mathbf{x}}\right)\right]^{\mathrm{T}} \boldsymbol{\Sigma}^{-1} \left[\left(\mathbf{z}_{d} - \bar{\mathbf{z}}\right) \right.\\&\left.\left.- \left(\mathbf{B A} + \mathbf{C}\right) \left(\mathbf{x}_{d} - \bar{\mathbf{x}}\right)\right]\right\}, \end{aligned}  $$

where

$$ \bar{\mathbf{x}} = \frac{1}{D}\sum_{d=1}^{D} \mathbf{x}_{d}, \qquad\qquad \bar{\mathbf{z}} = \frac{1}{D}\sum_{d=1}^{D} \mathbf{z}_{d}. $$

### Optimization

To optimize the objective function, there are many off-the-shelf *ℓ*_1_-penalized optimization tools. We use the Orthant-Wise Limited-memory Quasi-Newton (OWL-QN) algorithm described in [23]. The OWL-QN algorithm minimizes functions of the form

$$f(w) = loss(w) + c ||w||_{1}, $$

where *loss*(·) is an arbitrary differentiable loss function, and ||*w*||_1_ is the *ℓ*_1_-norm of the parameter vector. It is based on the L-BFGS Quasi-Newton algorithm [24], with modifications to deal with the fact that the *ℓ*_1_-norm is not differentiable. The algorithm is proven to converge to a local optimum of the parameter vector. The algorithm is very fast, and capable of scaling efficiently to problems with millions of parameters. Thus it is a good option for our problem where the parameter space is large when dealing with large scale eQTL data.

In addition to the loss function and penalized parameters, the OWL-QN algorithm also requires the gradient of the loss function, which (without detailed derivation) is given in the Additional file 1.

### Computational speedup

In this section, we discuss how to speedup the optimization process for the proposed model. In the previous section, we have shown that **A**, **B**, **C**, **W**, *σ*_1_, and *σ*_2_ are the parameters to be solved. Here, we first derive an updating scheme for *σ*_2_ when other parameters are fixed by following a similar technique as discussed in [25]. For other parameters, we develop an efficient method for calculating the inverse of the covariance matrix which is the main bottleneck of the optimization process.

#### Updating ***σ***_***2***_

When all other parameters are fixed, using spectral decomposition on $\left ({\sigma _{1}^{2}} \mathbf {B B}^{\mathrm {T}} + \mathbf {WW}^{\mathrm {T}}\right)$, we have

(12) $$ { \fontsize{9.5}{6}\begin{aligned} \boldsymbol{\Sigma} &= \left({\sigma_{1}^{2}} \mathbf{B B}^{\mathrm{T}} + \mathbf{WW}^{\mathrm{T}}\right) + {\sigma_{2}^{2}} \mathbf{I}_{N}\\ &= \left[\mathbf{U}, \mathbf{V}\right] \text{diag}\left(\lambda_{1}+{\sigma_{2}^{2}}, \ldots, \lambda_{N-q}+{\sigma_{2}^{2}}, 0, \ldots, 0\right) \left[\mathbf{U}, \mathbf{V}\right]^{\mathrm{T}}\\ &= \mathbf{U} \text{diag}\left(\lambda_{1}+{\sigma_{2}^{2}}, \ldots, \lambda_{N-q}+{\sigma_{2}^{2}}\right) \mathbf{U}^{\mathrm{T}}, \end{aligned}}  $$

where **U** is an *N*×(*N*−*q*) eigenvector matrix corresponding to the nonzero eigenvalues; **V** is an *N*×*q* eigenvector matrix corresponding to the zero eigenvalues. A reasonable solution should have no zero eigenvalues in ***Σ***, otherwise the loss function would be infinitely big. Therefore, *q*=0.

Thus

$$\boldsymbol{\Sigma}^{-1} = \mathbf{U} \text{diag}\left(\frac{1}{\lambda_{1}+{\sigma_{2}^{2}}}, \ldots, \frac{1}{\lambda_{N}+{\sigma_{2}^{2}}}\right) \mathbf{U}^{\mathrm{T}}. $$

Let **U** ^T^(**z**_*d*_−**B** **A** **x**_*d*_−**C** **x**_*d*_−***μ***_**B**_)= :[*η*_*d*,1_,*η*_*d*,2_,…,*η*_*d*,*N*_]^T^. Then solving *σ*_2_ is equivalent to minimizing

(13) $$  \begin{aligned} l\left({\sigma_{2}^{2}}\right) =&\, \frac{D \cdot N}{2} \log(2 \pi) + \frac{D}{2} \sum\limits_{s=1}^{N} \log\left(\lambda_{s}+{\sigma_{2}^{2}}\right) \\ &+ \frac{1}{2} \sum\limits_{d=1}^{D} \sum\limits_{s=1}^{N} \frac{\eta_{d, s}^{2}}{\lambda_{s}+{\sigma_{2}^{2}}}, \end{aligned}  $$

whose derivative is

$$l^{\prime}\left({\sigma_{2}^{2}}\right) = \frac{D}{2} \sum\limits_{s=1}^{N} \frac{1}{\lambda_{s}+{\sigma_{2}^{2}}} - \frac{1}{2} \sum_{d=1}^{D} \sum\limits_{s=1}^{N} \frac{\eta_{d, s}^{2}}{\left(\lambda_{s}+{\sigma_{2}^{2}}\right)^{2}}. $$

This is a 1-dimensional optimization problem that can be solved very efficiently.

#### Efficiently inverting the covariance matrix

From objective function Eq. 11 and the gradient of the parameters (given in the Additional file 1), the time complexity of each iteration in the optimization procedure is $\mathcal {O}(DN^{2}M+DN^{2}H+DN^{3}+DNMK)$. Since *M*≪*N* and *H*≪*N*, the third term of the time complexity ($\mathcal {O}(DN^{3})$) is the bottleneck of the overall performance. This is for computing the inverse of the covariance matrix

$$\boldsymbol{\Sigma} = {\sigma_{1}^{2}} \mathbf{BB}^{\mathrm{T}} + \mathbf{WW}^{\mathrm{T}} + {\sigma_{2}^{2}} \mathbf{I}_{N}, $$

which is much more time-consuming than other matrix multiplication operations.

We devise an acceleration strategy that calculates ***Σ*** ^−1^ using formula (14) in the following theorem. The complexity of computing the inverse reduces to $\mathcal {O}(M^{3}+H^{3})$.

##### **Theorem1**.

Given $\mathbf {B} \in \mathbb {R}^{N \times M}$, $\mathbf {W} \in \mathbb {R}^{N \times H}$, and

$$ \boldsymbol{\Sigma} = {\sigma_{2}^{2}} \mathbf{I}_{N} + {\sigma_{1}^{2}} \mathbf{BB}^{\mathrm{T}} + \mathbf{WW}^{\mathrm{T}}. $$

Then

(14) $$ \mathbf{\Sigma}^{-1} = \mathbf{T} - \mathbf{TWS}^{-1} \mathbf{W}^{\mathrm{T}} \mathbf{T},  $$

where

(15) $$ \mathbf{S} = \mathbf{I}_{H} + \mathbf{W}^{\mathrm{T}} \mathbf{TW},  $$

(16) $$ \mathbf{T} = \sigma_{2}^{-2}\left(\mathbf{I}_{N} - {\sigma_{1}^{2}} \mathbf{B} \left({\sigma_{2}^{2}}\mathbf{I}_{M} + {\sigma_{1}^{2}} \mathbf{B}^{\mathrm{T}}\mathbf{B}\right)^{-1} \mathbf{B}^{\mathrm{T}}\right).  $$

The proof is provided in the Additional file 1.

## Results and discussion

We apply our method to both simulation datasets and yeast eQTL datasets [26] to evaluate its performance. For simplicity, we refer to the proposed model that only considers group-wise associations as *Model 1*, and the model that considers both individual and group-wise associations as *Model 2*. For comparision, we select several recent eQTL methods, including LORS [27], MTLasso2G [14], FaST-LMM [11], SET-eQTL [13] and Lasso [3]. The tuning parameters in the selected methods are learned using cross-validation. All experiments are performed on a PC with 2.20 GHz Intel i7 eight-core CPU and 8 GB memory.

### Simulation study

**Figure 3:**
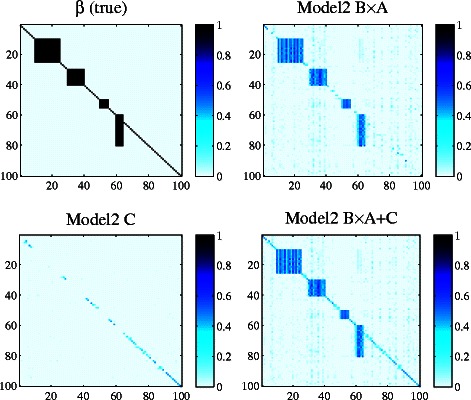
**Ground truth of matrix β and linkage weights estimated by Model 2 on simulated data.** The x-axis represents traits and y-axis represents SNPs. Normalized absolute values of regression coefficients are used. Darker color implies stronger association.

We first evaluate whether Model 2 can identify both individual and group-wise associations. We adopt a similar setup for simulation study to that in [27,28] and generate synthetic datasets as follows. 100 SNPs are randomly selected from the yeast eQTL dataset [26]. *N* gene expression profiles are generated by **Z**_*j*∗_=**β**_*j*∗_ **X**+*Ξ*_*j*∗_+**E**_*j*∗_ (1≤*j*≤*N*), where **E**_*j*∗_∼*N*(0,*η* *I*) (*η*=0.1) denotes Gaussian noise. *Ξ*_*j*∗_ is used to model non-genetic effects, which is drawn from *N*(**0**,*ρ* *Λ*), where *ρ*=0.1. *Λ* is generated by **F** **F** ^T^, where $\mathbf {F}\in \mathbb {R}^{D\times U}$ and **F**_*ij*_∼*N*(0,1). *U* is the number of hidden factors and is set to 10 by default. The association matrix **β** is shown in the top-left plot in Figure 3. The association strength is 1 for all selected SNPs. There are in total four group-wise associations of different scales. The associations on the diagonal are used to represent individual association signals in *cis*-regulation.

**Figure 4:**
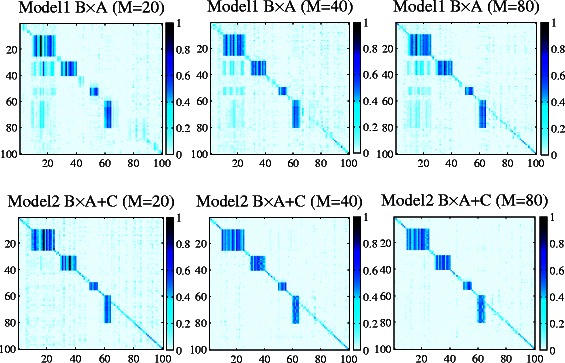
**Association weights estimated by Model 1 and Model 2 on simulated data with different** ***M*** **’s.** The x-axis represents traits and y-axis represents SNPs.

The remaining three plots in Figure 3 show associations estimated by Model 2. From the figure, we can see that Model 2 well captures both individual and group-wise signals. For comparison, Figure 4 visualizes the association weights estimated by Model 1 and Model 2 when varying the number of hidden variables (*M*). We observe that for Model 1, when *M*=20, most of the individual association signals on the diagonal are not captured. As *M* increases, more individual association signals are detected by Model 1. In contrast, Model 2 recovers both individual and group-wise linkage signals with small *M*.

Next, we generate 50 simulated datasets with different signal-to-noise ratios (defined as $SNR=\sqrt {\frac {Var(\mathbf {\beta }\mathbf {X})}{Var(\Xi + \mathbf {E})}}$) in the eQTL datasets [27] to compare the performance of the selected methods. Here, we fix *H*=10,*ρ*=0.1, and use different *η*’s to control *SNR*. For each setting, we report the averaged result from the 50 datasets. For the proposed methods, we use **B** **A**+**C** as the overall associations. Since FaST-LMM needs extra information (e.g., the genetic similarities between individuals) and uses PLINK format, we do not list it here and will compare it on the real data set.

**Figure 5:**
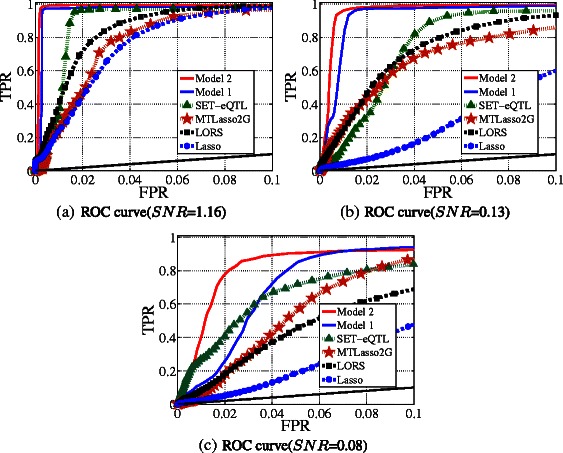
**The ROC curve of FPR-TPR on simulated data.** The black solid line denotes what random guessing would have achieved.

**Figure 6:**
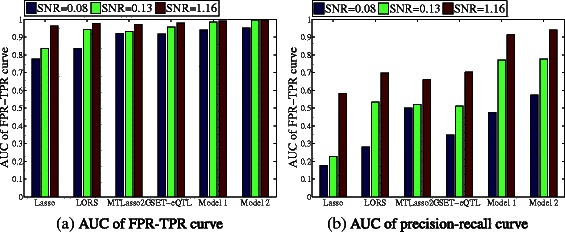
**The areas under the precision-recall/FPR-TPR curve (AUCs) of different methods with different signal-to-noise ratios (defined as** ***SNR*** **) on simulated data.**

Figure 5 shows the ROC curves of TPR-FPR for performance comparison. The corresponding areas under the TPR-FPR curve and the areas under the precision-recall curve (AUCs) [14] are shown in Figure 6. It can be seen that Model 2 outperforms all alternative methods by a large margin. Model 2 outperforms Model 1 because it considers both group-wise and individual associations. Model 1 outperforms SET-eQTL because it considers confounding factors that is not considered by SET-eQTL. SET-eQTL considers all associations as group-wise, thus it may miss some individual associations. MTLasso2G is comparable to LORS because MTLasso2G considers the group-wise associations while neglecting confounding factors. LORS considers the confounding factors, but does not distinguish individual and group-wise associations. LORS outperforms Lasso since confounding factors are not considered in Lasso.

#### Shrinkage of **C** and **B**×**A**

**Figure 7:**
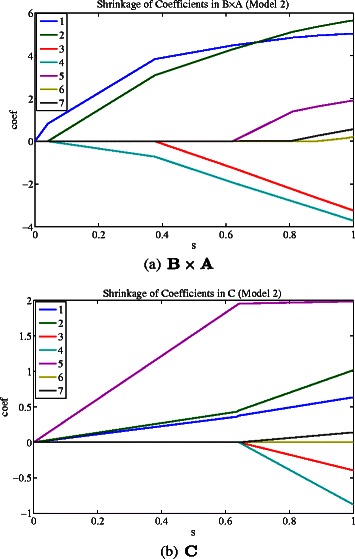
**Model 2 shrinkage of coefficients for** ***B×A*** **and** ***C*** **respectively.** Each curve represents a coefficient as a function of the scaled parameter $s=\frac {|\mathbf {B}\times \mathbf {A}|}{\max |\mathbf {B}\times \mathbf {A}|}$ or $s=\frac {|\mathbf {C}|}{\max |\mathbf {C}|}$.

As discussed in the Methods, the group-wise associations are encoded in **B**×**A** and individual associations are encoded in **C**. To enforce sparsity on **A**, **B** and **C**, we use Laplace prior on the elements of these matrices. Thus, it is interesting to study the the overall shrinkage of **B**×**A** and **C**. We randomly generate 7 predictors ({ *x*_1_ **,** *x*_2_ **,** **…** **,** *x*_7_}) and 1 response (**z**) with sample size 100. *x*_*i*_∼*N*(**0**,0.6·**I**)(*i*∈[ 1,7]). The response vector was generated with the formula: $\mathbf {z}=5\cdot (\mathbf {x_{1}}+\mathbf {x_{2}})-3\cdot (\mathbf {x_{3}}+\mathbf {x_{4}})+2\cdot \mathbf {x_{5}}+\tilde {\boldsymbol {\epsilon }}$ and $\tilde {\boldsymbol {\epsilon }}\in N(\mathbf {0},\mathbf {I})$. Thus, there are two groups of predictors ({ *x*_1_,*x*_2_} and { *x*_3_,*x*_4_}) and one individual predictor *x*_5_. Figure 7 shows the Model 2 shrinkage of coefficients for **B**×**A** and **C** respectively. Each curve represents a coefficient as a function of the scaled parameter $s=\frac {|\mathbf {B}\times \mathbf {A}|}{\max |\mathbf {B}\times \mathbf {A}|}$ or $s=\frac {|\mathbf {C}|}{\max |\mathbf {C}|}$. We can see that the two groups of predictors can be identified by **B**×**A** as the most important variables, and the individual predictor can be identified by **C**.

#### Computational efficiency evaluation

**Figure 8:**
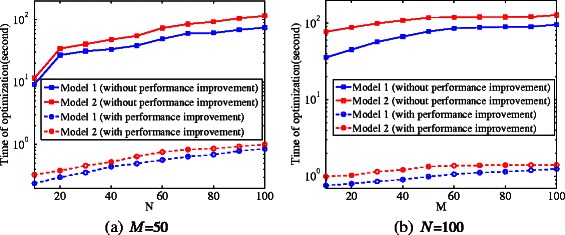
**Running time performance on simulated data when varying** ***N*** **and** ***M*** **.** When varying *N*, we fix *M*, and when varying *M*, we fix *N*.

Scalability is an important issue for eQTL study. To evaluate the techniques for speeding up the computational efficiency, we compare the running time with/without these techniques. Figure 8 shows the running time when varying the number of hidden variables (*M*) and number of traits (*N*). The results are consistent with the theoretical analysis in Methods part that the time complexity is reduced to $\mathcal {O}(M^{3}+H^{3})$ from $\mathcal {O}(N^{3})$ when using the improved method for inverting the covariance matrix. We also observe that Model 2 uses slightly more time than Model 1, since it has more parameters to optimize. However, to get similar performance, Model 1 needs a significantly larger number of hidden variables *M*. As shown in Figure 8(a), a larger *M* results in a longer running time. In some cases, Model 2 is actually faster than Model 1. As an example, to obtain the same performance (i.e., AUC), Model 1 needs 60 hidden variables (*M*), while Model 2 only needs *M*=20. In this case, from Figure 8(a), we can observe that Model 2 needs less time than Model 1 to obtain the same results.

### Yeast eQTL study

We apply the proposed methods to a yeast (Saccharomyces cerevisiae) eQTL dataset of 112 yeast segregants generated from a cross of two inbred strains [26]. The dataset originally includes expression profiles of 6229 gene expression traits and genotype profiles of 2956 SNP markers. After removing SNPs with more than 10% missing values and merging consecutive SNPS with high linkage disequilibrium, we obtain 1017 SNPs with distinct genotypes [29]. In total, 4474 expression profiles are selected after removing the ones with missing values. It takes about 5 hours for Model 1, and 3 hours for Model 1 to run to completion. The regularization parameters are set by grid search in {0.1, 1, 10, 50, 100, 500, 1000, 2000}. Specifically, grid search trains the model with each combinations of three regularization parameters in the grid and evaluates their performance (by measuring out-of-sample loss function value) for a two-fold cross validation. Finally, the grid search algorithm outputs the settings that achieved the smallest loss in the validation procedure.

**Figure 9:**
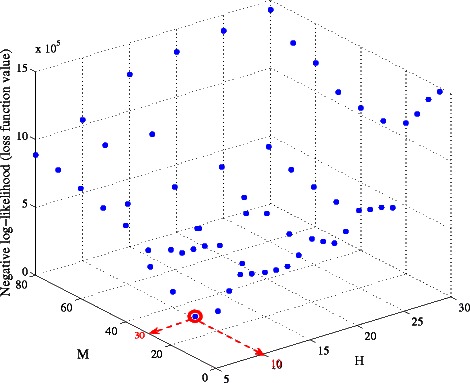
**Parameter tuning for** ***M*** **and** ***H*** **(Model 2).**

We use hold-out validation to find the optimal number of hidden variables *M* and *H* for each model. Specifically, we partition the samples into 2 subsets of equal size. We use one subset as training data and test the learned model using the other subset of samples. By measuring out-of-sample predictions, we can find optimal combination of *M* and *H* that avoids over-fitting. For each combination, optimal values for regularization parameters were determined with two-fold cross validation. The loss function values for different {*M*, *H*} combinations of Model2 are shown in Figure 9. We find that *M* = 30 and *H* = 10 for Model 2 delivers the best overall performance. Similarly, we find that the optimal *M* and *H* values for Model 1 are 150 and 10 respectively.

**Figure 10:**
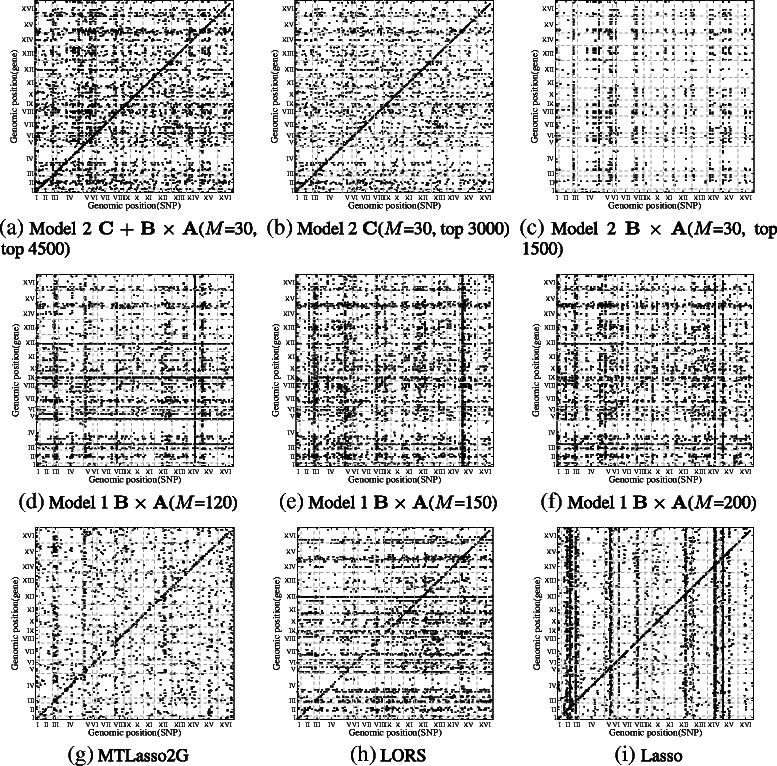
**Significant associations discovered by different methods in yeast.** The top 4500 associations ranked by abs(weight) are shown in each plot unless otherwise noted. The x-axis represents SNPs and y-axis represents genes (traits). Both SNPs and genes are arranged by their locations in the genome. **C**+**B**×**A** and **C** of Model 2 have stronger *cis*-regulatory signals and weaker *trans*-regulatory bands than Model 1, LORS, and Lasso. **C** of Model 2 has weaker *trans*-regulatory bands than others. LORS has weaker *trans*-regulatory bands than Lasso since it considers confounding factors.

The significant associations given by Model 1, Model 2, LORS, MTLasso2G and Lasso are shown in Figure 10. For Model 2, we can clearly see that the estimated matrices **C** and **B**×**A** well capture the non group-wise and group-wise signals respectively. **C**+**B**×**A** and **C** of Model 2 have stronger *cis*-regulatory signals and weaker *trans*-regulatory bands than that of Model 1, LORS, and Lasso. **C** of Model 2 has the weakest *trans*-regulatory bands. LORS has weaker *trans*-regulatory bands than Lasso since it considers confounding factors. With more hidden variables (larger *M*), Model 1 obtains stronger *cis*-regulatory signals.

#### cis- and trans-enrichment analysis

In total, the proposed two methods detect about 6000 associations with non-zero weight values (**B**×**A** for Model 1 and **C**+**B**×**A** for Model 2). We estimate their FDR values by following the method proposed in [27]. With FDR ≤ 0.01, both models obtain about 4500 associations. The visualization of significant associations detected by different methods is provided in Additional file 1.

We apply *cis-* and *trans-*enrichment analysis on the discovered associations. In particular, we follow the standard *cis*-enrichment analysis [30,31] to compare the performance of two competing models. The intuition behind *cis*-enrichment analysis is that more *cis*-acting SNPs are expected than *trans*-acting SNPs. A two-step procedure is used in the *cis*-enrichment analysis [30]: (1) for each model, we apply a one-tailed Mann-Whitney test on each SNP to test the null hypothesis that the model ranks its *cis* hypotheses (we use <500 bp for yeast) no better than its *trans* hypotheses, (2) for each pair of models compared, we perform a two-tailed paired Wilcoxon sign-rank test on the *p*-values obtained from the previous step. The null hypothesis is that the median difference of the *p*-values in the Mann-Whitney test for each SNP is zero. The *trans*-enrichment is implemented using a similar strategy as in [32], in which genes regulated by transcription factors^c^ are used as *trans*-acting signals.

The results of pairwise comparison of selected models are shown in Table 2. A *p*-value shows how significant a method on the left column outperforms a method in the top row in terms of *cis*-enrichment or *trans*-enrichment. We observe that the proposed Model 2 has significantly better *cis*-enrichment scores than other methods. For *trans*-enrichment, Model 2 is the best, and FaST-LMM comes in second. This is because both Model 2 and FaST-LMM consider confounding factors (FaST-LMM considers confounders from population structure) and joint effects of SNPs, but only Model 2 considers grouping of genes. Model 1 has poor performance because a larger *M* may be needed for Model 1 to capture those individual associations.

**Table 2:** **Pairwise comparison of different models using** ***cis*** **-enrichment and** ***trans*** **-enrichment in yeast**

***cis*** **-enrichment**		**FaST-LMM**	**C of Model 2**	**MTLasso2G**	**B × A of Model 1**	**LORS**	**Lasso**
	**C**+**B**×**A** of Model 2	0.4351	<0.0001	<0.0001	<0.0001	<0.0001	<0.0001
	FaST-LMM	-	0.2351	<0.0001	<0.0001	<0.0001	<0.0001
	**C**of Model 2	-	-	0.0221	<0.0001	<0.0001	<0.0001
	MTLasso2G	-	-	-	<0.0001	<0.0001	<0.0001
	**B**×**A**of Model 1	-	-	-	-	<0.0001	<0.0001
	LORS	-	-	-	-	-	0.0052
*trans*-enrichment		**B**×**A** of Model 2	FaST-LMM	MTLasso2G	LORS	**B**×**A** of Model 1	Lasso
	**C**+**B**×**A** of Model 2	0.4245	0.3123	0.0034	0.0029	0.0027	0.0023
	**B**×**A** of Model 2	-	0.3213	0.0132	0.0031	0.0028	0.0026
	FaST-LMM	-	-	0.0148	0.0033	0.0031	0.0029
	MTLasso2G	-	-	-	0.0038	0.0037	0.0032
	LORS	-	-	-	-	0.0974	0.0151
	**B**×**A**of Model 1	-	-	-	-	-	0.0564

A *p*-value shows how significant a method on the left column outperforms a method in the top row in terms of *cis*-enrichment or *trans*-enrichment.

#### Reproducibility of trans regulatory hotspots between studies

We also evaluate the consistency of calling eQTL hotspots between two independent glucose yeast datasets [33]. The glucose environment from Smith et al. [33] shares a common set of segregants. It includes 5493 probes measured in 109 segregates. Since our algorithm aims at finding group-wise associations, we focus on the consistency of regulatory hotspots.

We examine the reproducibility of *trans* regulatory hotspots based on the following criteria [18,19,27]. For each SNP, we count the number of associated genes from the detected SNP-gene associations. We use this number as the regulatory degree of each SNP. For Model2, LORS, and Lasso, all SNP-Gene pairs with non-zero association weights are defined as associations. Note that Model2 uses **B** **A**+**C** as the overall associations. For FaST-LMM, SNP-Gene pairs with a *q*-value < 0.001 are defined as associations. Note that we also tried different cutoffs for FaST-LMM (from 0.01 to 0.001), the results are similar. SNPs with large regulatory degrees are often referred to as hotspots. We sort SNPs by the extent of *trans* regulation (regulatory degrees) in a descending order. We denote the sorted SNPs lists as *S*_1_ and *S*_2_ for the two yeast datasets. Let ${S_{1}^{T}}$ and ${S_{2}^{T}}$ be the top *T* SNPs in the sorted SNP lists. The trans calling consistency of detected hotspots is defined as $\frac {|{S_{1}^{T}}\bigcap {S_{2}^{T}}|}{T}$.

**Figure 11:**
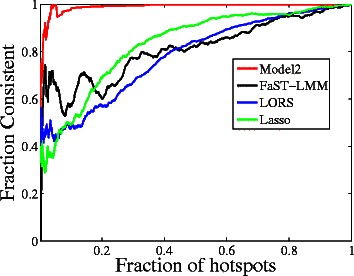
**Consistency of detected eQTL hotspots between two independent yeast eQTL datasets.**

Figure 11 compares the reproducibility of *trans* regulatory hotspots given by different studies. It can be seen that the proposed Model2 gives much higher consistency than any other competitors do. In particular, the consistency of *trans* hotspots suggests the superiority of Model2 in identifying hotspots that are likely to have a true genetic underpinning.

#### Gene ontology enrichment analysis

As discussed in Methods, hidden variables **y** in the middle layer may model the joint effect of SNPs that have influence on a group of genes. To better understand the learned model, we look for correlations between a set of genes associated with a hidden variable and GO categories (Biological Process Ontology) [34]. In particular, for each gene set *G*, we identify the GO category whose set of genes is most correlated with *G*. We measure the correlation by a *p*-value determined by the Fisher’s exact test. Since multiple gene sets *G* need to be examined, the raw *p*-values need to be calibrated because of the multiple testing problem [35]. To compute the calibrated *p*-values for each gene set *G*, we perform a randomization test, wherein we apply the same test to randomly created gene sets that have the same number of genes as *G*. Specifically, the enrichment test is performed using DAVID [29]. And gene sets with calibrated *p*-values less than 0.01 are considered as significantly enriched.

The results from Model 2 are reported in Table 3. Each row of Table 3 represents the gene set associated with a hidden variable. All of these detected gene sets are significantly enriched in certain GO categories. The significantly enriched gene sets of Model 1 are included in Additional file 1.

**Table 3:** **Summary of all detected groups of genes from Model 2 on yeast data**

^***a***^ **Group ID**	^***b***^ **SNPs set size**	^***c***^ **gene set size**	^***d***^ **GO category**
1	63	294	oxidation-reduction process ^∗^
2	78	153	thiamine biosynthetic process ^∗^
3	94	871	rRNA processing ^∗∗∗^
4	64	204	nucleosome assembly ^∗∗^
5	70	288	ATP synthesis coupled proton transport ^∗∗∗^
6	43	151	branched chain family amino acid biosynthetic... ^∗∗^
7	76	479	mitochondrial translation ^∗∗∗^
8	47	349	transmembrane transport ^∗∗^
9	64	253	cytoplasmic translation ^∗∗∗^
10	72	415	response to stress ^∗∗^
11	64	225	mitochondrial translation ^∗^
12	62	301	oxidation-reduction process ^∗∗^
13	83	661	oxidation-reduction process ^∗^
14	69	326	cytoplasmic translation ^∗^
15	71	216	oxidation-reduction process ^∗^
16	66	364	methionine metabolic process ^∗^
17	74	243	cellular amino acid biosynthetic process ^∗∗∗^
18	63	224	transmembrane transport ^∗∗^
19	23	50	de novo’ pyrimidine base biosynthetic process ^∗^
20	66	205	cellular amino acid biosynthetic process ^∗∗∗^
21	81	372	oxidation-reduction process ^∗∗^
22	33	126	oxidation-reduction process ^∗∗∗^
23	81	288	pheromone-dependent signal transduction... ^∗∗^
24	53	190	pheromone-dependent signal transduction... ^∗∗^
25	91	572	oxidation-reduction process ^∗∗∗^
26	66	46	cellular cell wall organization ^∗^
27	111	1091	translation ^∗∗∗^
28	89	362	cellular amino acid biosynthetic process ^∗∗^
29	62	217	transmembrane transport ^∗∗^
30	71	151	cellular aldehyde metabolic process ^∗∗^

^***a***^Group ID corresponding to Figure 12. ^***b***^Number of SNPs in the group. ^***c***^Number of genes in the group. ^***d***^The most significant GO category enriched in the associated gene set. The enrichment test was performed using DAVID [29]. The gene function is defined by GO category. Adjusted *p*-values are reported by using permutation test. Adjusted *p*-values are indicated by ∗, where ^∗^10^−2^∼10^−3^, ^∗∗^10^−3^∼10^−5^, ^∗∗∗^10^−5^∼10^−10^.

**Figure 12:**
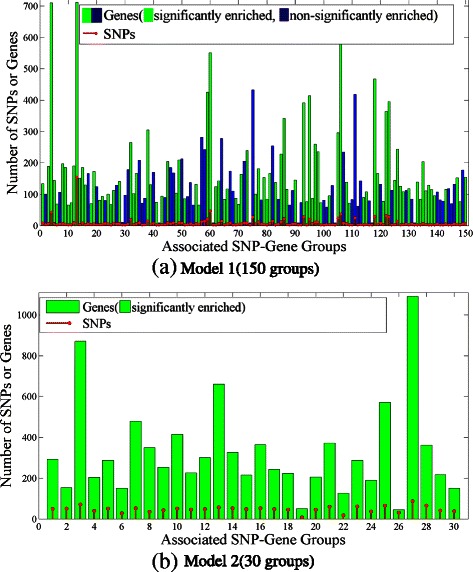
**Number of SNPs and genes in each group-wise association detected by Model 1 and Model 2 in yeast.** x-axis is the associated SNP-Gene Group ID, y-axis is the number of SNPs or genes in the group. The red line denotes the number of SNPs in the group and the bar denotes number of genes in the group, among which the green bar indicates that the group of genes is significantly enriched by some GO categrory, while the red bar indicates that the groups of genes is not significantly enriched.

For comparison, we visualize the number of SNPs and genes in each group-wise association in Figure 12. We observe that 90 out of 150 gene sets reported by Model 1 are significantly enriched, and all 30 gene sets reported by Model 2 are significantly enriched (GOA results of Model 1 are reported in Additional file 2). This indicates that Model 2 is able to detect group-wise linkages more precisely than Model 1. We also study the hotspots detected by LORS, which affect >10 gene traits [28]. Specifically, we delve into the top 15 hotspots detected by LORS (ranking by number of associated genes for each SNP), as listed in Table 4. We can see that only 9 out of 15 top ranked hotspots are significantly enriched.

**Table 4:** **Summary of the top 15 detected hotspots by LORS**

**chr**	**start**	**end**	**size**	**GO category**	**adjusted p-value**
XII	659357	662627	36	sterol biosynthetic process	7.18E-05
XII	1056097	1056097	31	telomere maintenance via recombination	4.72E-08
**XV**	**154177**	**154309**	**29**	**amino acid catabolic process to alcohol via Ehrlich pathway**	**0.052947053**
III	201166	201167	23	regulation of mating-type specific transcription, DNA-dependent	0.001998002
**XV**	**143597**	**150651**	**23**	**response to stress**	**0.672327672**
III	81832	92391	22	pheromone-dependent signal transduction involved in conjugation with cellular fusion	1.76E-03
VIII	111682	111690	22	cell adhesion	0.002947528
IX	139462	139512	21	cellular response to nitrogen starvation	0.00106592
**XV**	**170945**	**180961**	**20**	**cell adhesion**	**0.053946054**
III	105042	105042	19	branched chain family amino acid biosynthetic process	5.51357E-08
**XIII**	**46070**	**46084**	**19**	**cell adhesion**	**0.050949051**
XV	563943	563943	19	transport	0.003996004
**I**	**41483**	**42639**	**18**	**cellular response to nitrogen starvation**	**0.016983017**
III	175799	177850	18	pheromone-dependent signal transduction involved in conjugation with cellular fusion	7.47E-03
**I**	**36900**	**37068**	**17**	**signal transduction**	**0.547452547**

Bold groups are not significantly enriched.

## Conclusion

A crucial challenge in eQTL study is to understand how multiple SNPs interact with each other to jointly affect the expression level of genes. In this paper, we propose a sparse graphical model to identify novel group-wise eQTL associations. The proposed model can also take into account potential confounding factors and individual associations. *ℓ*_1_-regularization is applied to learn the sparse structure of the graphical model. We also introduce computational techniques to make this approach suitable for large scale studies. Extensive experimental evaluations using both simulated and real datasets demonstrate that the proposed methods can effectively capture both individual and group-wise signals and significantly outperform the state-of-the-art eQTL mapping methods.

## Endnotes

^a^ The software is implemented in both C++ and matlab, and publicly available at http://www.cs.unc.edu/~weicheng/Group-Wise-EQTL.zip.

^b^ For example, 0, 1, 2 may encode the homozygous major allele, heterozygous allele, and homozygous minor allele, respectively.

^c^ http://www.yeastract.com/download.php.

## Additional files

Additional file 1
**Results of GO enrichment test for significantly enriched groups of genes detected by Model 1.**

Additional file 2
**Proof of Theorem 1.**

## References

[1] Bochner BR (2003). New technologies to assess genotype henotype relationships. Nat Rev Genet..

[2] Michaelson J, Loguercio S, Beyer A (2009). Detection and interpretation of expression quantitative trait loci (eQTL). Methods..

[3] Tibshirani R (1996). Regression shrinkage and selection via the lasso. J Royal Statist Soc B..

[4] Cheung VG, Spielman RS, Ewens KG, Weber TM, Morley M, Burdick JT (2005). Mapping determinants of human gene expression by regional and genome-wide association. Nature..

[5] Musani SK, Shriner D, Liu N, Feng R, Coffey CS, Yi N, Tiwari HK, Allison DB (2007). Detection of gene x gene interactions in genome-wide association studies of human population data. Human Heredity..

[6] Pujana MA, Han J-DJ, Starita LM, Stevens KN, Muneesh Tewari EA (2007). Network modeling links breast cancer susceptibility and centrosome dysfunction. Nat Genet..

[7] Lander ES (2011). Initial impact of the sequencing of the human genome. Nature..

[8] Holden M, Deng S, Wojnowski L, Kulle B (2008). GSEA-SNP: applying gene set enrichment analysis to SNP data from genome-wide association studies. Bioinformatics..

[9] Wu MC, Lee S, Cai T, Li Y, Boehnke M, Lin X (2011). Rare-variant association testing for sequencing data with the sequence kernel association test. Am J Hum Genet..

[10] Braun R, Buetow K (2011). Pathways of distinction analysis: a new technique for multi-SNP analysis of GWAS data. PLoS Genet..

[11] Listgarten J, Lippert C, Kang EY, Xiang J, Kadie CM, Heckerman D (2013). A powerful and efficient set test for genetic markers that handles confounders. Bioinformatics..

[12] Huang Y, Wuchty S, Ferdig MT, Przytycka TM (2009). Graph theoretical approach to study eqtl: a case study of plasmodium falciparum. ISMB..

[13] Cheng W, Zhang X, Wu Y, Yin X, Li J, Heckerman D, Wang W (2012). Inferring novel associations between snp sets and gene sets in eqtl study using sparse graphical model. ACM-BCB..

[14] Chen X, Shi X, Xu X, Wang Z, Mills R, Lee C, Xu J. A two-graph guided multi-task lasso approach for eqtl mapping. In: Lawrence ND, Girolami MA, editors. Proceedings of the Fifteenth International Conference on Artificial Intelligence and Statistics (AISTATS) ’12. vol. 22: 2012. p. 208–217.

[15] Cheng W, Zhang X, Guo Z, Shi Y, Wang W (2014). Graph regularized dual lasso for robust eqtl mapping. Bioinformatics.

[16] Gao C, Brown CD, Engelhardt BE. A latent factor model with a mixture of sparse and dense factors to model gene expression data with confounding effects. ArXiv e-prints. 2013.

[17] Leek JT, Storey JD (2007). Capturing heterogeneity in gene expression studies by surrogate variable analysis. PLoS Genet..

[18] Joo JW, Sul JH, Han B, Ye C, Eskin E (2014). Effectively identifying regulatory hotspots while capturing expression heterogeneity in gene expression studies. Genome Biol..

[19] Fusi N, Stegle O, Lawrence ND (2012). Joint modelling of confounding factors and prominent genetic regulators provides increased accuracy in genetical genomics studies. PLoS Comput Biol..

[20] Carlos M, Carvalhoa JELJRNQW, West M, Jeffrey Changa (2008). High-Dimensional Sparse Factor Modeling: Applications in Gene Expression Genomics. J Am Stat Assoc..

[21] Lee S-I, Dudley AM, Drubin D, Silver PA, Krogan NJ, Pe’er D, Koller D (2009). Learning a prior on regulatory potential from eqtl data. PLoS Genet..

[22] Ng A. Feature selection, l1 vs. l2 regularization, and rotational invariance. In: Proceedings of the International Conference on Machine Learning (ICML): 2004.

[23] Andrew G, Gao J. Scalable training of l1-regularized log-linear models. In: Proceedings of the Twenty-Fourth International Conference on Machine Learning (ICML): 2007.

[24] Nocedal J, Wright SJ (1999). Numerical optimization.

[25] Kang HM, Zaitlen NA, Wade CM, Kirby A, Heckerman D, Daly MJ, Eskin E (2008). Efficient control of population structure in model organism association mapping. Genetics..

[26] Rachel B, Brem JW, John DStorey, Kruglyak L (2005). Genetic interactions between polymorphisms that affect gene expression in yeast. Nature..

[27] Yang C, Wang L, Zhang S, Zhao H (2013). Accounting for non-genetic factors by low-rank representation and sparse regression for eQTL mapping. Bioinformatics..

[28] Lee S, Xing EP (2012). Leveraging input and output structures for joint mapping of epistatic and marginal eQTLs. Bioinformatics..

[29] Huang DAW, Sherman BT, Lempicki RA (2009). Systematic and integrative analysis of large gene lists using DAVID bioinformatics resources. Nat Protoc..

[30] Listgarten J, Kadie C, Schadt EE, Heckerman D (2010). Correction for hidden confounders in the genetic analysis of gene expression. Proc Natl Acad Sci USA..

[31] McClurg P, Janes J, Wu C, Delano DL, Walker JR, Batalov S, Takahashi JS, Shimomura K, Kohsaka A, Bass J, Wiltshire T, Su AI (2007). Genomewide association analysis in diverse inbred mice: power and population structure. Genetics..

[32] Yvert G, Brem RB, Whittle J, Akey JM, Foss E, Smith EN, Mackelprang R, Kruglyak L (2003). Trans-acting regulatory variation in Saccharomyces cerevisiae and the role of transcription factors. Nat Genet..

[33] Smith EN, Kruglyak L (2008). Gene-environment interaction in yeast gene expression. PLoS Biol..

[34] The Gene Ontology Consortium (2000). Gene ontology: tool for the unification of biology. Nat Genet..

[35] Westfall PH, Young SS. Resampling-based multiple testing; 1993.

