# Supplementary material for: Fast and robust group-wise eQTL mapping using sparse graphical models
Source: BMC Bioinformatics. 2015 Jan 16;16:2. doi: 10.1186/s12859-014-0421-z (PMC4387667; doi:10.1186/s12859-014-0421-z)
Supplement: Additional file 1 — Results of GO enrichment test for significantly enriched groups of genes detected by Model 1. [file 12859_2014_421_MOESM1_ESM.pdf]

RESEARCH

[Supplementary Information] Fast and Robust Group-Wise eQTL Mapping Using Sparse Graphical Models

Wei Cheng[1], Shi Yu[2], Xiang Zhang[3] and Wei Wang[4*]

*Correspondence: weiwang@cs.ucla.edu
[4]Department of Computer Science, University of California, Los Angeles, 3531-G Boelter Hall, CA 90095 Los Angeles, USA
Full list of author information is available at the end of the article

Preparation for Derivatives of $\mathcal{O}$ for Model 2

For notational simplicity, we denote

$$\mathbf{t}_d = (\mathbf{z}_d - \bar{\mathbf{z}}) - (\mathbf{BA} + \mathbf{C})(\mathbf{x}_d - \bar{\mathbf{x}}),$$

$$\mathbf{\Psi}_d = \frac{1}{2}(\mathbf{\Sigma}^{-1} - \mathbf{\Sigma}^{-1}\mathbf{t}_d\mathbf{t}_d{}^{\mathrm{T}}\mathbf{\Sigma}^{-1}).$$

Derivative with respect to σ_1

$$\nabla_{\sigma_1}\mathcal{O} = 2\sigma_1\sum_{d=1}^{D}\{\mathrm{tr}[\mathbf{\Psi}_d]\mathbf{BB}^{\mathrm{T}}\}.$$

Derivative with respect to σ_2

$$\nabla_{\sigma_2}\mathcal{O} = 2\sigma_2\sum_{d=1}^{D}\{\mathrm{tr}[\mathbf{\Psi}_d]\}.$$

Derivative with respect to $\mathbf{A}$

$$\nabla_{\mathbf{A}}\mathcal{O} = -\sum_{d=1}^{D}[\mathbf{B}^{\mathrm{T}}\mathbf{\Sigma}^{-1}\mathbf{t}_d(\mathbf{x}_d - \bar{\mathbf{x}})^{\mathrm{T}}].$$

Derivative with respect to $\mathbf{B}$

$$\nabla_{\mathbf{B}}\mathcal{O} = \mathbf{\Xi_1} + \mathbf{\Xi_2},$$

where

$$\mathbf{\Xi_1} = -\sum_{d=1}^{D}[\mathbf{\Sigma}^{-1}\mathbf{t}_d(\mathbf{x}_d - \bar{\mathbf{x}})^{\mathrm{T}}\mathbf{A}^{\mathrm{T}}],$$

$$(\mathbf{\Xi_2})_{ij} = \sigma_1^2 \sum_{d=1}^{D} \{\mathrm{tr}[\mathbf{\Psi}_d(\mathbf{E}_{ij}\mathbf{B}^{\mathrm{T}} + \mathbf{B}\mathbf{E}_{ji})]\}.$$

($\mathrm{tr}[\cdot]$ stands for trace; $\mathbf{E}_{ij}$ is the single-entry matrix: 1 at (i, j) and 0 elsewhere.)

We speed up this calculation by exploiting sparsity of $\boldsymbol{E}_{ij}$ and $\mathrm{tr}[\cdot]$. (The following equation uses *Einstein summation convention* to better illustrates the idea.)

$$\begin{aligned}
(\mathbf{\Xi}_2)_{ij} &= \sigma_1^2 \sum_{d=1}^{D} \{\mathrm{tr}[\mathbf{\Psi}_d(\boldsymbol{E}_{ij}\boldsymbol{B}^T + \boldsymbol{B}\boldsymbol{E}_{ji})]\} && (1)\\
&= \sigma_1^2 \sum_{d=1}^{D} \{\mathrm{tr}[\mathbf{\Psi}_d\boldsymbol{E}_{ij}\boldsymbol{B}^T + \mathbf{\Psi}_d\boldsymbol{B}\boldsymbol{E}_{ji}]\} && (2)\\
&= \sigma_1^2 \sum_{d=1}^{D} \{\mathrm{tr}[(\mathbf{\Psi}_d)^k_l(\boldsymbol{E}_{ij})^l_m(\boldsymbol{B}^T)^m_n + (\mathbf{\Psi}_d)^k_l(\boldsymbol{B})^l_m(\boldsymbol{E}_{ji})^m_n]\} && (3)\\
&= \sigma_1^2 \sum_{d=1}^{D} \{(\mathbf{\Psi}_d)^k_l(\boldsymbol{E}_{ij})^l_m(\boldsymbol{B}^T)^m_k + (\mathbf{\Psi}_d)^k_l(\boldsymbol{B})^l_m(\boldsymbol{E}_{ji})^m_k\} && (4)\\
&= \sigma_1^2 \sum_{d=1}^{D} \{(\mathbf{\Psi}_d)^k_i(\boldsymbol{B}^T)^j_k + (\mathbf{\Psi}_d)^i_l(\boldsymbol{B})^l_j\} && (5)\\
&= \sigma_1^2 \sum_{d=1}^{D} \{\sum_{k=1}^{N}[(\mathbf{\Psi}_d)_{k,i}(\boldsymbol{B}^T)_{j,k}] + \sum_{l=1}^{N}[(\mathbf{\Psi}_d)_{i,l}(\boldsymbol{B})_{l,j}]\} && (6)\\
&= \sigma_1^2 \sum_{d=1}^{D} \{\sum_{k=1}^{N}[(\boldsymbol{B}^T)_{j,k}(\mathbf{\Psi}_d)_{k,i}] + \sum_{l=1}^{N}[(\mathbf{\Psi}_d)_{i,l}(\boldsymbol{B})_{l,j}]\}. && (7)
\end{aligned}$$

Therefore,

$$\begin{aligned}
\mathbf{\Xi}_2 &= \sigma_1^2 \sum_{d=1}^{D} [(\boldsymbol{B}^T\mathbf{\Psi}_d)^T + \mathbf{\Psi}_d\boldsymbol{B}] && (8)\\
&= \sigma_1^2 \sum_{d=1}^{D} [\mathbf{\Psi}_d^T\boldsymbol{B} + \mathbf{\Psi}_d\boldsymbol{B}] && (9)\\
&= 2\sigma_1^2 \sum_{d=1}^{D} \mathbf{\Psi}_d\boldsymbol{B}. && (10)
\end{aligned}$$

Derivative with respect to $\mathbf{C}$

$$\nabla_{\mathbf{C}}\mathcal{O} = -\sum_{d=1}^{D} [\mathbf{\Sigma}^{-1}\mathbf{t}_d(\mathbf{x}_d - \bar{\mathbf{x}})^{\mathrm{T}}].$$

Derivative with respect to $\mathbf{W}$

$$\nabla_{\mathbf{W}}\mathcal{O} = \sum_{d=1}^{D} \{\mathrm{tr}[\mathbf{\Psi}_d(\mathbf{E}_{ij}\mathbf{W}^{\mathrm{T}} + \mathbf{W}\mathbf{E}_{ji})]\} = 2\sum_{d=1}^{D} \mathbf{\Psi}_d\mathbf{W}.$$

Proof of Theorem 1

Before giving the formal proof for Theorem 1, we first introduce Lemma 1, which follows from the definition of matrix inverse.

Lemma 1 *For all* $\mathbf{P} \in \mathbb{R}^{N\times M}$*, if* $\mathbf{I}_M + \mathbf{P}^{\mathrm{T}}\mathbf{P}$ *is invertible, then*

$$(\mathbf{I}_N + \mathbf{P}\mathbf{P}^{\mathrm{T}})^{-1} = \mathbf{I}_N - \mathbf{P}(\mathbf{I}_M + \mathbf{P}^{\mathrm{T}}\mathbf{P})^{-1}\mathbf{P}^{\mathrm{T}}.$$

Here we provide a more general proof, which can be modified to derive more involved cases.

Proof 1 *We denote*

$$\mathbf{Q} = \sigma_2^2\mathbf{I}_N + \sigma_1^2\mathbf{B}\mathbf{B}^{\mathrm{T}}, \tag{11}$$

that is,

$$\mathbf{\Sigma} = \sigma_2^2\mathbf{I}_N + \sigma_1^2\mathbf{B}\mathbf{B}^{\mathrm{T}} + \mathbf{W}\mathbf{W}^{\mathrm{T}} = \mathbf{Q} + \mathbf{W}\mathbf{W}^{\mathrm{T}}. \tag{12}$$

By Lemma 1, we have

$$\mathbf{Q}^{-1} = \mathbf{T} = \sigma_2^{-2}(\mathbf{I}_N - \sigma_1^2\mathbf{B}(\sigma_2^2\mathbf{I}_M + \sigma_1^2\mathbf{B}^{\mathrm{T}}\mathbf{B})^{-1}\mathbf{B}^{\mathrm{T}}).$$

$\mathbf{Q}$ *is symmetric positive definite, hence its inverse,* $\mathbf{T}$*, is symmetric positive definite. Since every symmetric positive definite matrix has exactly one symmetric positive definite square root, we can write*

$$\mathbf{T} = \mathbf{R}\mathbf{R},$$

where $\mathbf{R}$ *is an* $N \times N$ *symmetric positive definite matrix.*

It is clear that, $\mathbf{Q} = \mathbf{T}^{-1} = (\mathbf{R}\mathbf{R})^{-1} = \mathbf{R}^{-1}\mathbf{R}^{-1}$*, which leads to* $\mathbf{R}\mathbf{Q}\mathbf{R} = \mathbf{R}\mathbf{R}^{-1}\mathbf{R}^{-1}\mathbf{R} = \mathbf{I}_N$*, and therefore*

$$\mathbf{R}\mathbf{\Sigma}\mathbf{R} = \mathbf{I}_N + \mathbf{R}\mathbf{W}\mathbf{W}^{\mathrm{T}}\mathbf{R} = \mathbf{I}_N + \mathbf{R}\mathbf{W}\mathbf{W}^{\mathrm{T}}\mathbf{R}^{\mathrm{T}}.$$

Note that the above and the following formulas follow the fact that $\mathbf{R}$ *is symmetric.*

Once again, by Lemma 1, we have

$$(\mathbf{R}\mathbf{\Sigma}\mathbf{R})^{-1} = \mathbf{I}_N - \mathbf{R}\mathbf{W}\mathbf{S}^{-1}\mathbf{W}^{\mathrm{T}}\mathbf{R}^{\mathrm{T}},$$

where

$$\mathbf{S} = \mathbf{I}_H + \mathbf{W}^{\mathrm{T}}\mathbf{R}^{\mathrm{T}}\mathbf{R}\mathbf{W} = \mathbf{I}_H + \mathbf{W}^{\mathrm{T}}\mathbf{T}\mathbf{W}.$$

Therefore,

$$\mathbf{\Sigma}^{-1} = \mathbf{R}(\mathbf{R}\mathbf{\Sigma}\mathbf{R})^{-1}\mathbf{R} = \mathbf{R}\mathbf{R} - \mathbf{R}\mathbf{R}\mathbf{W}\mathbf{S}^{-1}\mathbf{W}^{\mathrm{T}}\mathbf{R}^{\mathrm{T}}\mathbf{R},$$

and thus

$$\mathbf{\Sigma}^{-1} = \mathbf{T} - \mathbf{T}\mathbf{W}\mathbf{S}^{-1}\mathbf{W}^{\mathrm{T}}\mathbf{T}$$

Author details

[1]Department of Computer Science, UNC at Chapel Hill, 201 S Columbia St., NC 27599 Chapel Hill, USA. [2]Department of Mathematics, University of Science and Technology of China, 443 Huangshang Rd, 230026 Hefei, China. [3]Department of Elect. Eng. and Computer Science, Case Western Reserve University, 10900 Euclid Avenue, OH 44106 Cleveland, USA. [4]Department of Computer Science, University of California, Los Angeles, 3531-G Boelter Hall, CA 90095 Los Angeles, USA.

References
