# Supplementary material for: Fast and robust group-wise eQTL mapping using sparse graphical models
Source: BMC Bioinformatics. 2015 Jan 16;16:2. doi: 10.1186/s12859-014-0421-z (PMC4387667; doi:10.1186/s12859-014-0421-z)
Supplement: Additional file 2 — Proof of Theorem 1. [file 12859_2014_421_MOESM2_ESM.pdf]

RESEARCH

[Supplementary Information] Fast and Robust Group-Wise eQTL Mapping Using Sparse Graphical Models

Wei Cheng[1], Shi Yu[2], Xiang Zhang[3] and Wei Wang[4*]

[*]Correspondence: weiwang@cs.ucla.edu
[4]Department of Computer Science, University of California, Los Angeles, 3531-G Boelter Hall, CA 90095 Los Angeles, USA
Full list of author information is available at the end of the article

Results of GO enrichment test for significantly enriched groups of genes detected by Model 1

Author details
[1]Department of Computer Science, UNC at Chapel Hill, 201 S Columbia St., NC 27599 Chapel Hill, USA. [2]Department of Mathematics, University of Science and Technology of China, 443 Huangshang Rd, 230026 Hefei, China. [3]Department of Elect. Eng. and Computer Science, Case Western Reserve University, 10900 Euclid Avenue, OH 44106 Cleveland, USA. [4]Department of Computer Science, University of California, Los Angeles, 3531-G Boelter Hall, CA 90095 Los Angeles, USA.

References

1. Huang, d.a.W., Sherman, B.T., Lempicki, R.A.: Systematic and integrative analysis of large gene lists using DAVID bioinformatics resources. Nat Protoc **4**(1), 44–57 (2009)

^{a}Group ID	^{b}SNPs set size	^{c}gene set size	^{d}GO category
1	8	134	branched chain family amino acid biosynthetic process**
3	6	189	oxidation-reduction process***
4	43	710	cytoplasmic translation***
5	6	144	ion transport*
6	2	69	arginine biosynthetic process*
8	6	197	cellular amino acid biosynthetic process**
9	4	185	transmembrane transport*
10	2	66	cellular response to nitrogen starvation*
11	2	73	cellular response to nitrogen starvation*
12	9	191	pheromone-dependent signal transduction involved in conjugation with cellular fusion*
13	154	712	cytoplasmic translation***
14	3	151	amino acid catabolic process to alcohol via Ehrlich pathway*
15	8	185	oxidation-reduction process**
16	3	130	arginine biosynthetic process*
18	3	70	arginine biosynthetic process*
19	5	173	cellular amino acid biosynthetic process*
21	3	81	cellular aldehyde metabolic process*
22	4	93	cellular amino acid biosynthetic process**
24	5	101	iron ion homeostasis*
25	2	67	cellular amino acid metabolic process**
26	7	112	oxidation-reduction process*
28	6	141	oxidation-reduction process*
32	19	265	cellular amino acid biosynthetic process*
33	3	102	glycogen biosynthetic process*
34	6	166	oxidation-reduction process*
38	15	305	cellular amino acid biosynthetic process***
39	4	131	telomere maintenance via recombination**
41	2	75	cellular response to nitrogen starvation*
43	3	94	cellular response to nitrogen starvation*
45	9	205	cellular amino acid biosynthetic process*
48	3	104	telomere maintenance via recombination*
49	10	210	oxidation-reduction process*
51	2	86	cellular aldehyde metabolic process*
55	6	132	cytogamy*
56	4	66	cellular cell wall organization*
59	21	425	methionine biosynthetic process*
60	46	551	cellular amino acid biosynthetic process**
62	2	124	ion transport**
63	6	143	iron ion homeostasis*
65	2	84	cellular response to nitrogen starvation*
66	5	117	transposition, RNA-mediated*
69	2	88	one-carbon metabolic process*
70	4	68	cellular response to nitrogen starvation*
71	5	164	oxidation-reduction process*
73	8	240	cellular amino acid biosynthetic process**
76	5	101	cellular response to nitrogen starvation*
77	12	181	mitochondrial electron transport, ubiquinol to cytochrome c***
79	4	153	cellular amino acid biosynthetic process**
80	2	85	hexose transport*
81	7	166	oxidation-reduction process**
83	2	137	cellular amino acid biosynthetic process*
85	12	228	cellular amino acid biosynthetic process***
86	22	342	cellular amino acid biosynthetic process*
87	3	116	cellular amino acid biosynthetic process*
90	6	146	hexose transport*

Table 1 Summary of detected groups of genes that are significantly enriched from Model 1 (Part I). ^{a}Group ID corresponding to Fig. 9. ^{b}Number of SNPs in the group. ^{c}Number of genes in the group. ^{d}The most significant GO category enriched in the associated gene set. The enrichment test was performed using DAVID ([1]). The gene function is defined by GO category. Adjusted p-values are reported by using permutation test. Adjusted p-values are indicated by $*$, where $*10^{-2} \sim 10^{-3}$, $**10^{-3} \sim 10^{-5}$,$***10^{-5} \sim 10^{-10}$.

^{a}Group ID	^{b}SNPs set size	^{c}gene set size	^{d}GO category
93	28	391	ATP synthesis coupled proton transport**
94	2	76	oxidation-reduction process**
95	20	414	nucleosome assembly*
96	6	87	cellular response to nitrogen starvation*
97	11	260	oxidation-reduction process*
98	11	236	mitochondrial electron transport, ubiquinol to cytochrome c*
99	2	73	cellular response to nitrogen starvation*
102	3	95	cellular aldehyde metabolic process**
105	24	296	cellular amino acid biosynthetic process**
106	37	651	oxidation-reduction process***
108	6	138	oxidation-reduction process*
109	2	72	siderophore transport*
114	2	90	amino acid transmembrane transport*
115	4	108	arginine biosynthetic process*
118	30	467	cellular amino acid biosynthetic process***
119	4	166	methionine biosynthetic process**
121	3	77	iron ion homeostasis*
122	31	364	cellular amino acid biosynthetic process***
123	29	395	cellular amino acid biosynthetic process***
125	3	145	cellular amino acid biosynthetic process**
126	14	244	cellular response to nitrogen starvation*
127	2	126	cellular amino acid biosynthetic process*
128	3	108	telomere maintenance via recombination*
130	2	118	oxidation-reduction process*
133	2	139	cell adhesion*
134	2	84	cell adhesion*
135	6	204	oxidation-reduction process**
136	3	111	arginine biosynthetic process*
137	2	129	response to pheromone**
138	2	115	transmembrane transport*
139	2	95	cellular aldehyde metabolic process*
143	5	116	cellular amino acid biosynthetic process*
147	4	152	mitochondrial electron transport, ubiquinol to cytochrome c**
148	2	76	cellular aldehyde metabolic process**
150	5	154	fermentation*

Table 2 Summary of detected groups of genes that are significantly enriched from Model 1 (Part II). ^{a}Group ID corresponding to Fig. 9. ^{b}Number of SNPs in the group. ^{c}Number of genes in the group. ^{d}The most significant GO category enriched in the associated gene set. The enrichment test was performed using DAVID ([1]). The gene function is defined by GO category. Adjusted p-values are reported by using permutation test. Adjusted p-values are indicated by $*$, where $*10^{-2} \sim 10^{-3}$, $**10^{-3} \sim 10^{-5}$,$***10^{-5} \sim 10^{-10}$.
